# Gut microbial characteristics in poor appetite and undernutrition: a cohort of older adults and microbiota transfer in germ‐free mice

**DOI:** 10.1002/jcsm.13002

**Published:** 2022-06-14

**Authors:** Kristina S. Fluitman, Mark Davids, Louise E. Olofsson, Madelief Wijdeveld, Valentina Tremaroli, Bart J.F. Keijser, Marjolein Visser, Fredrik Bäckhed, Max Nieuwdorp, Richard G. IJzerman

**Affiliations:** ^1^ Department of Internal Medicine Amsterdam University Medical Centers, location VUmc Amsterdam The Netherlands; ^2^ Amsterdam Public Health Research Institute Amsterdam The Netherlands; ^3^ Department of Vascular Medicine Amsterdam University Medical Centers, location AMC Amsterdam The Netherlands; ^4^ Wallenberg Laboratory, Department of Molecular and Clinical Medicine, Institute of Medicine University of Gothenburg Gothenburg Sweden; ^5^ Department of Microbiology and Systems Biology TNO Healthy Living Zeist The Netherlands; ^6^ Department of Preventive Dentistry, Academic Center for Dentistry Amsterdam University of Amsterdam and VU University Amsterdam The Netherlands; ^7^ Department of Health Sciences, Faculty of Science Vrije Universiteit Amsterdam The Netherlands; ^8^ Novo Nordisk Foundation Center for Basic Metabolic Research, Faculty of Health and Medical Sciences University of Copenhagen Copenhagen Denmark; ^9^ Department of Clinical Physiology Region Västra Götaland, Sahlgrenska University Hospital Gothenburg Sweden

**Keywords:** Gut microbiota, Undernutrition, Appetite, Older adults, Microbiota transfer experiment, Germ‐free mice

## Abstract

**Background:**

Older adults are particularly prone to the development of poor appetite and undernutrition. Possibly, this is partly due to the aged gut microbiota. We aimed to evaluate the gut microbiota in relation to both poor appetite and undernutrition in community‐dwelling older adults. Furthermore, we studied the causal effects of the microbiota on body weight and body composition by transferring faecal microbiota from cohort participants into germ‐free mice.

**Methods:**

First, we conducted a cross‐sectional cohort study of 358 well‐phenotyped Dutch community‐dwelling older adults from the Longitudinal Aging Study Amsterdam. Data collection included body measurements, a faecal and blood sample, as well as extensive questionnaires on appetite, dietary intake, and nutritional status. Appetite was assessed by the Council of Nutrition Appetite Questionnaire (CNAQ) and undernutrition was defined by either a low body mass index (BMI) (BMI < 20 kg/m^2^ if <70 years or BMI < 22 kg/m^2^ if ≥70 years) or >5% body weight loss averaged over the last 2 years. Gut microbiota composition was determined with 16S rRNA sequencing. Next, we transferred faecal microbiota from 12 cohort participants with and without low BMI or recent weight loss into a total of 41 germ‐free mice to study the potential causal effects of the gut microbiota on host BMI and body composition.

**Results:**

The mean age (range) of our cohort was 73 (65–93); 58.4% was male. Seventy‐seven participants were undernourished and 21 participants had poor appetite (CNAQ < 28). A lower abundance of the genus *Blautia* was associated with undernutrition (log2 fold change = −0.57, Benjamini–Hochberg‐adjusted *P* = 0.008), whereas higher abundances of taxa from Lachnospiraceae, Ruminococcaceae UCG‐002, *Parabacteroides merdae* , and *Dorea formicigenerans* were associated with poor appetite. Furthermore, participants with poor appetite or undernutrition had reduced levels of faecal acetate (*P* = 0.006 and 0.026, respectively). Finally, there was a trend for the mice that received faecal microbiota from older adults with low BMI to weigh 1.26 g less after 3 weeks (*P* = 0.086) and have 6.13% more lean mass (in % body weight, *P* = 0.067) than the mice that received faecal microbiota from older adults without low BMI or recent weight loss.

**Conclusions:**

This study demonstrates several associations of the gut microbiota with both poor appetite and undernutrition in older adults. Moreover, it is the first to explore a causal relation between the aged gut microbiota and body weight and body composition in the host. Possibly, microbiota‐manipulating strategies will benefit older adults prone to undernutrition.

## Introduction

Older adults are at increased risk of developing undernutrition, a condition of macronutrient depletion resulting from inadequate protein or energy intake.^1^ Undernutrition affects roughly 10–30% of older Europeans and is associated with increased morbidity and mortality.^2^ The development of undernutrition in older adults is largely explained by the age‐related decline in appetite, called ‘Anorexia of Aging’.^3^ The aged gut microbiota is thought to play an important role in anorexia of aging^4^ and the subsequent development of undernutrition, but evidence is scarce.

The gut microbiota consists of trillions of bacteria that have colonized the human gut and interact with their human host in various ways.^5^ They ferment otherwise indigestible polysaccharides and proteins and produce various metabolites, such as short‐chain fatty acids (SCFAs). SCFAs interact with host metabolism and influence appetite as part of the gut–brain axis.^5^ Age‐related compositional changes in the gut microbiota could result in a dysbiotic, pro‐inflammatory gut microbial profile with reduced capacity for energy harvest, predisposing an older individual to undernutrition.^6^, ^7^, ^8^ Moreover, some associations of the gut microbial composition with chronic inflammation,^6^ poor appetite,^4^ sarcopenia,^9^ and frailty^10^ have been demonstrated. However, most studies are observational, and therefore, causality has not been sufficiently demonstrated.

We hypothesize that the gut microbiota and metabolite profiles differ between older adults with and without poor appetite or undernutrition and that the gut microbiota can causally affect weight and body composition in its host. To test our hypotheses, we first completed a cross‐sectional cohort study in 358 Dutch community‐dwelling older adults. We then transferred faecal microbiota from 12 cohort participants with and without undernutrition into germ‐free mice to evaluate the microbiota‐induced effects on body weight, body composition, and food intake. If the aged gut microbiota contributes to anorexia of aging and the development of undernutrition, specific microbiota‐manipulating strategies could be developed to promote a healthy microbial profile and prevent undernutrition in older adults.

## Methods

### Human cohort

This cross‐sectional cohort study is embedded within the ongoing Longitudinal Aging Study Amsterdam (LASA). The designs of the current study^11^ and the LASA study^12^ are described in more detail elsewhere. In total, 1642 LASA participants who had body measurements taken during the last LASA data‐collection wave (2015/16) were pre‐screened for inclusion based on available LASA data. Eligible participants (*n* = 727) were asked to participate and screened on remaining inclusion and exclusion criteria by phone. For 360 participants, home visits were performed between 2017 and 2018. During the visit, informed consent was signed and all data were collected. One participant was excluded because no accurate body measurements could be obtained, and one because no viable faecal sample was obtained. Inclusion criteria for this study were age over 65 years, valid measurement of body weight in the most recent LASA examination wave (2015/16), community‐dwelling, and living in the Netherlands. Exclusion criteria were over‐nutrition [i.e. body mass index (BMI) above 30 kg/m^2^ or >2% body weight gain between the latest two LASA examination waves (2011/13 to 2015/16)], diagnosed active malignancy, mini‐mental state examination (MMSE) score < 18, or antibiotic use in the previous 3 months. This study was approved by the medical ethics committee of the Amsterdam UMC, location VUmc, and carried out in accordance with the 1964 Declaration of Helsinki and its later amendments.

### Clinical measurements

Body weight was measured wearing only undergarments. Some wore indoor clothing without shoes, in which case 1 kg was subtracted. BMI (kg/m^2^) was calculated using the earliest LASA height measurement available. Undernutrition was defined as low BMI (BMI < 20 kg/m^2^ if age < 70 years or BMI < 22 kg/m^2^ if ≥70 years)^1^ or more than 5% body weight loss averaged over 2 years. Body composition was measured by body impedance analysis (BIA) using the Bodystat 1500MDD device (Bodystat Ltd., Isle of Man, UK). Appendicular skeletal muscle mass index (ASMMI) was calculated using the formula by Sergi *et al*., which was specifically developed for Caucasian community‐dwelling older adults (intra‐class correlation with dual X‐ray absorptiometry measured ASMM was 0.961, standard error of estimate (SEE) 1.1 kg, *P* < 0.001).^13^ Blood pressure was measured at the left arm in duplicate using an automatic Omron M7 device (Omron Corporation, Tokyo, Japan). Calf and mid‐upper arm circumference were measured in duplicate as well.

Appetite was assessed by the eight‐item Council of Nutrition Appetite Questionnaire, its score ranging from 8 (worst) to 40 (best). A score < 28 was used to define poor appetite.^14^ Frequency and average amount of foods consumed over the past 4 weeks were determined with the 238‐item Dutch version of the Food Frequency Questionnaire (FFQ).^15^ From the FFQ data, energy intake (kcal/day), macronutrient intake [percentage of daily energy intake (En%)], and the Mediterranean Diet Score (MDS) were calculated. A higher MDS indicates better adherence to a typically healthy, Mediterranean diet (total score range 0–55).^16^

Taste function was assessed with taste strips (Burghart Messtechnik GmbH, Wedel, Germany). The test consists of taste strips that were impregnated with increasing concentrations of sweet, sour, salty, bitter, and umami tastes.^17^ The total score ranges from 0 to 20 and a score < 6 was considered poor. Smell function was assessed with Sniffin' Sticks identification test (score 0–16) (Burghart Messtechnik GmbH). A score ≤ 9 was considered poor. Participants were asked not to smoke, chew gum, eat, or drink anything other than water in the hour preceding the sensory tests. Data on socio‐demographics, smoking status, depression symptoms [Centre for Epidemiologic Studies Depression (CESD) score],^18^ medication use, and cognitive status (MMSE score^19^) were obtained from the previous LASA examination wave (2015/16).

### Mouse experiment

Three faecal microbiota donor groups were made: low BMI (LBMI, six donors), weight loss (WL, five donors), and healthy controls (HC, six donors). Participants from the human cohort were selected as donor without a priori knowledge of their microbial composition if they had <2% weight fluctuations or a BMI of 24–27 kg/m^2^ (HC) or were at the extremes of LBMI or WL within our study cohort. Donor samples were selected without a priori knowledge of microbial composition. Age and sex of the donors were equally distributed across groups. Donor samples were prepared for donation as described elsewhere.^20^ Male germ‐free Swiss Webster mice, aged 10 to 11 weeks, were colonized by oral gavage with 200 μL of the donor inoculum after a 5 h fast on Day 1 and Day 3 of the experiment. They were housed in individually ventilated cages (ISOcage N system, Tecniplast, Buguggiate, Italy), with a maximum of four mice per cage. All mice from the same cage received donor inoculum from one donor. Where possible, littermates from the LBMI and WL mice were used as HC. Water and autoclaved chow food were given *ad libitum* throughout the experiment. All food given and all leftovers were weighted to determine food intake per cage. This was averaged over the combined body mass of the mice in that cage (g/g). Body weight measurements were done weekly. Fat mass and lean mass (the latter not including free water, bone mass, and hair of claws) were measured with an EchoMRI instrument after the second gavage and at the end of the 3 week experiment. Investigators were not blinded to group allocation. The donor inoculum and the Week 3 faecal sample were analysed using 16S rRNA sequencing. The mouse experiment protocols were approved by the local animal ethics committee at the University of Gothenburg, Sweden.

### Biosampling and analysis

Participants were asked to collect two stool samples in sterile containers (Sarstedt). One sample was kept at room temperature, transported to the research facility, aliquoted in portions of 100–150 mg (for 16S rRNA sequencing) and 500 mg (for the mouse experiment), and stored in a −80°C freezer within 36 h of production. 16S rRNA sequencing was carried out as described earlier at the Wallenberg Laboratory (Sahlgrenska Academy at University of Gothenburg, Sweden).^21^ Total genomic DNA was extracted from the 100–150 mg aliquots using a repeated bead beating method as previously described.^22^ The V4 region of the 16S rRNA gene was sequenced on a MiSeq system (RTA Version 1.17.28, bundled with MCS Version 2.5; Illumina) with 515F and 806R primers designed for dual indexing^23^ and the V2 kit (2 × 250 bp paired‐end reads; Illumina). 16S rRNA genes from each sample were amplified in volumes of 25 μL containing 1 × 5 PRIME HotMasterMix (5 PRIME), 200 nM of each primer, 0.4 mg/ml BSA, 5% dimethylsulfoxide, and 20 ng of genomic DNA. PCR was carried out under the following conditions: initial denaturation for 3 min at 94°C; followed by 25 cycles of denaturation for 45 s at 94°C; annealing for 60 s at 52°C; elongation for 90 s at 72°C; and a final elongation step for 10 min at 72°C. PCR products were purified with the NucleoSpin Gel and PCR Clean‐Up kit (Macherey‐Nagel) and quantified using the Quant‐iT PicoGreen dsDNA kit (Invitrogen). Purified PCR products were diluted to 10 ng/μL and pooled in equal amounts. The pooled amplicons were purified again using Ampure magnetic purification beads (Agencourt) to remove short amplification products. Positive and negative DNA extraction controls, as well as positive PCR controls, were included in analysis. Amplicon reads were merged and processed using USEARCH.^24^ Merged reads with expected error rates higher than 1 were filtered after which sequence variants (SVs) were inferred using UNOISE.^25^ The unfiltered reads were used to determine the SV abundances. Taxonomy was assigned using the RDP classifier^26^ and SILVA^27^ 16S ribosomal database V132.

The other faecal sample (for SCFA determination) was kept in the participant's refrigerator as SCFAs are more volatile and then transported to the research facility in a cool pack where it was homogenized, aliquoted in portions of 200–300 mg, and stored at −80°C within 36 h of production. Faecal SCFA levels were measured using high‐performance liquid chromatography (HPLC) with UV detection according to the method of De Baere *et al*.^28^ Dry weights were determined after freeze‐drying a homogenized faecal aliquot for 24 h. The raw SCFA HPLC measurements were corrected for the difference in the wet and dry weight for each sample.

Blood samples were taken during the home visit after a 3 h fast and centrifuged immediately at 1800 *g* at room temperature. Plasma was aliquoted and stored at −80°C. Plasma metabolite analyses were carried out as described earlier^29^ by Metabolon (Durham, North Carolina), using ultra‐HPLC coupled to tandem mass spectrometry. Then, each biochemical was rescaled to set the median equal to 1. Missing values, generally due to the sample measurement falling below the limit of detection, were then imputed with the minimum observed value. One non‐centrifuged blood sample was directly brought to the hospital laboratory for conventional blood count and HbA1c determination. Venapuncture was performed in 149 participants as some participants thought it was too burdensome and not all researchers conducting the home visits were certified to perform venapuncture.

### Statistical analysis

All microbiota and metabolite‐related analyses were performed in R (Version 4.0.0). Particularly, the Phyloseq package,^30^ Vegan package,^31^ DESeq2 package,^32^ and ggplot2 package^33^ were used for analysis and visualization. Participant characteristics and covariates were depicted as mean ± standard deviation, median and interquartile ranges, or number and percentages as appropriate. The overall microbiota composition was first evaluated using Bray–Curtis dissimilarity. All variables that explained a significant amount of Bray–Curtis dissimilarity were identified with PERMANOVA. Next, correlations among these variables were studied with Spearman's correlation analysis. To identify important confounding and/or mediation covariates, the PERMANOVAs for poor appetite and undernutrition were adjusted one by one for each variable that (1) explained a significant amount of variance in Bray–Curtis dissimilarity and (2) was significantly correlated to either poor appetite or undernutrition. All covariates that were identified as possible confounders by negating the association of poor appetite or undernutrition with Bray–Curtis dissimilarity were subsequently included as covariates in the following microbiota analyses (i.e. alpha‐diversity and DESeq analyses). Next, alpha‐diversity was calculated using species richness, Shannon index, and Faith's phylogenetic diversity index. Associations of poor appetite and undernutrition with the alpha‐diversity were assessed with linear regression models (crude and adjusted). Then, differential abundant taxa for poor vs. normal appetite, and undernutrition vs. no undernutrition were assessed with DESeq analysis (crude and adjusted). Log2 fold change in bacterial abundance was reported. Only taxa with a mean abundance > 10 were considered. Correlation between microbiome and metabolite profiles was tested using the protest function implemented in the vegan package with 99 999 permutations. PCoA ordinations of the Euclidean distance of clr transformed metabolite data and Bray–Curtis dissimilarity of the microbiome were compared. Generalized linear models (GLMs) (crude and adjusted) were used to test differences in metabolites between poor and normal appetite, and undernutrition and no undernutrition. Associations of poor appetite and undernutrition with the composition of faecal propionate, acetate, and butyrate were multivariately tested using MANOVA. Associations with absolute concentrations of each SCFA individually were tested with Bonferroni‐corrected Student's *t*‐test. Differences among donor characteristics, donor microbiota alpha‐diversity, or mouse baseline characteristics were tested with Kruskal–Wallis or Fisher's exact test. Pairwise testing with Bonferroni correction was applied if significant differences were found. Differences in Bray–Curtis dissimilarity among donor groups were tested with PERMANOVA. Differences in weight (g body weight) of the mice among groups were tested with linear mixed models with a random intercept for donor and mouse ID, and adjusted for baseline values, using SPSS software Version 22 (SPSS Inc., Chicago, Illinois). Differences in lean and fat mass (in g or % body weight) were tested similarly, but without a random intercept for mouse ID. Differences in food intake (g/g body weight of mice/cage) were tested similarly, but without adjustment for baseline values. Associations of faecal mouse microbial Bray–Curtis dissimilarity with mouse phenotype were tested with PERMANOVA, only including one random mouse per donor as co‐housed mice from the same donor can be considered microbial replicates.^34^ For both the DESeq and GLM, correction for multiple testing was done by the Benjamini–Hochberg procedure and a *P*‐value < 0.01 was considered statistically significant. For all other analyses, a *P*‐value < 0.05 was considered statistically significant.

## Results

### Participant characteristics

A total of 358 well‐phenotyped Dutch community‐dwelling older adults from LASA^12^ was included in this cross‐sectional cohort. Participants were on average 73 years old (ranging from 65 to 93 years), and 209 (58.4%) were male. Seventy‐seven (21.5%) participants were undernourished, either based on LBMI (*n* = 40) or WL (*n* = 43). Poor appetite was present in 21 participants (5.9%). Poor appetite occurred alongside undernutrition in nine participants. A full overview of participant characteristics is depicted in Supporting Information, *Table* S1.

### Alterations in the gut microbial composition are associated with poor appetite and undernutrition

In our cohort, 4235 unique bacterial 16S markers were identified. All data have been rarified to 24 410 reads. Firmicutes were most abundant, followed by Bacteroidetes, Actinobacteria, and Proteobacteria (*Figure* S1).

**Figure 1:**
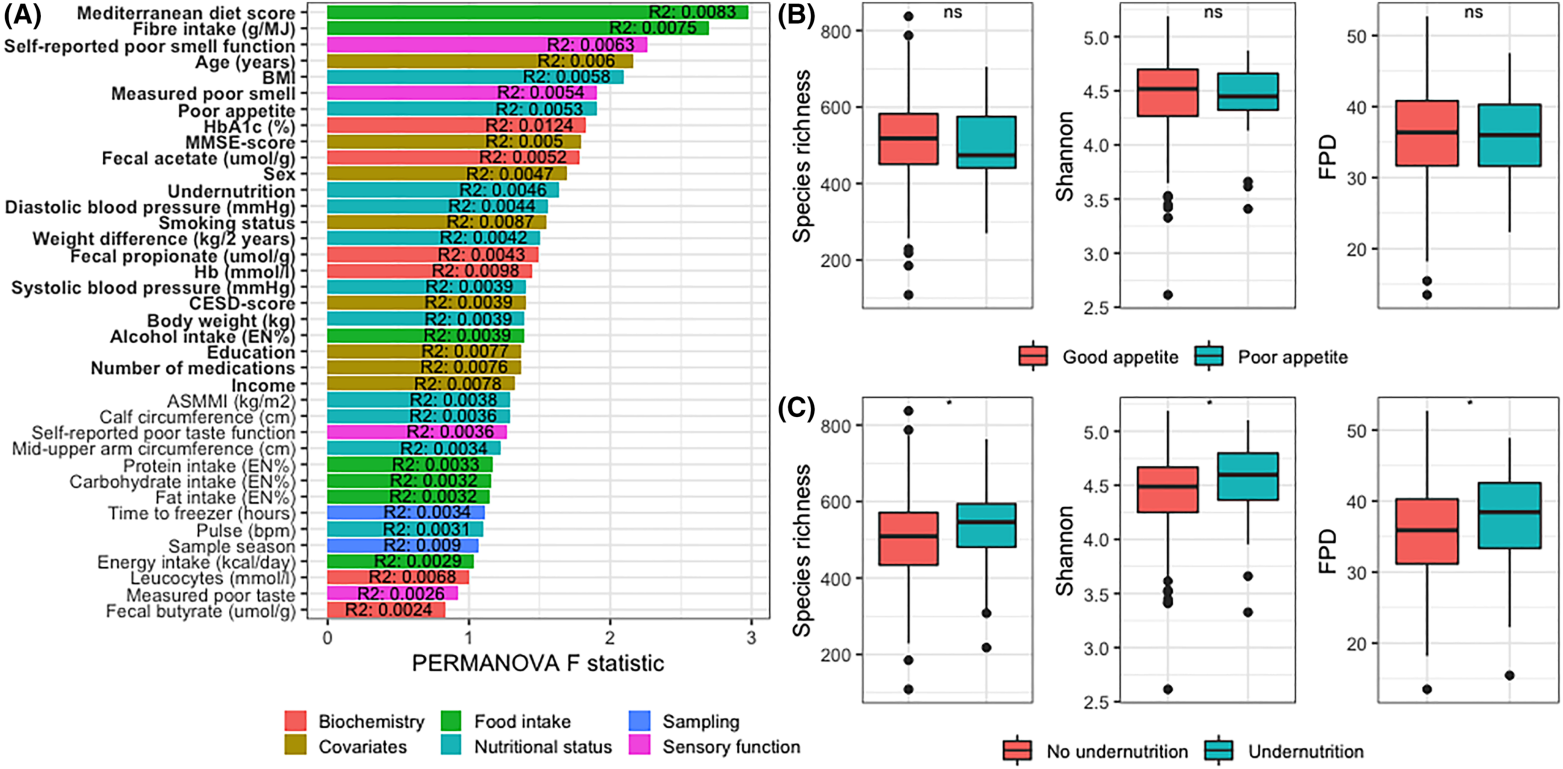
Variables associated with alpha‐diversity and beta‐diversity in the human cohort. *(A)* Barplot showing the *F*‐statistic of the PERMANOVA model for each factor. Bars are coloured based on the type of variable. The *R* ^2^ is noted in each bar, depicting the variance in Bray–Curtis beta‐diversity explained by each variable. Bold text indicated statistical significance (*P* < 0.05). *(B* and *C)* Boxplots of alpha‐diversity measures species richness, Shannon index, and FPD for participants with or without poor appetite *(B)* and with or without undernutrition *(C)*. Boxplots show median (centre line), interquartile ranges (boxes), 1.5× the interquartile ranges (whiskers), and outliers. Differences in alpha‐diversity were tested with linear regression models, adjusted for age and Mediterranean Diet Score. ASMMI, appendicular skeletal muscle mass index; CESD, Centre for Epidemiologic Studies Depression; FPD, Faith's phylogenetic diversity; MMSE, mini‐mental state examination.

We first analysed the overall microbial composition by calculating Bray–Curtis dissimilarity as measure for beta‐diversity (i.e. inter‐individual microbiota compositional dissimilarity). Both poor appetite and undernutrition univariately explained a significant amount of variance in Bray–Curtis dissimilarity (PERMANOVA *R* ^2^ = 0.0053, *P* = 0.004, and *R* ^2^ = 0.0046, *P* = 0.011, respectively), as did several other variables (*Figure* 1A). Of these covariates, poor appetite was correlated with lower MDS and fibre intake, poor self‐reported smell, older age, lower MMSE score, higher CESD score, lower faecal acetate levels, smoking, less alcohol intake, lower education, polypharmacy, and lower income. Undernutrition was correlated with poor appetite, older age, lower faecal acetate levels, sex, lower diastolic and systolic blood pressure, lower Hb, and lower income (*Figure* S2). To test if any of these covariates confounded the microbiota–appetite or microbiota–undernutrition relationships, we adjusted the PERMANOVA models on poor appetite and undernutrition for each of these. The association between beta‐diversity and poor appetite only became non‐significant after adjusting for MDS (MDS‐adjusted PERMANOVA *R* ^2^ = 0.0037, *P* = 0.06). Similarly, the association with undernutrition became non‐significant after adjustment for age (age‐adjusted PERMANOVA *R* ^2^ = 0.004, *P* = 0.06). Other covariates, such as sex or education, did not significantly alter the relation of poor appetite or undernutrition with Bray–Curtis dissimilarity. Because we identified MDS and age as important modulators of the microbiota–appetite and microbiota–undernutrition relationships, the subsequent microbiota analyses were adjusted for age, and both age and MDS.

Next, we evaluated alpha‐diversity (i.e. intra‐individual microbiota diversity). Alpha‐diversity indices were calculated using species richness, Shannon index, and Faith's phylogenetic diversity index. Participants with undernutrition, but not with poor appetite, had significantly higher values for all three alpha‐diversity measures, both in crude and adjusted linear regression models (species richness: *B*_adjusted_ = 30.4, *P* = 0.034; Shannon index: *B*_adjusted_ = 0.1, *P* = 0.014; and Faith's phylogenetic diversity: *B*_adjusted_ = 2.0, *P* = 0.022) (*Figure* 1B and 1C).

**Figure 2:**
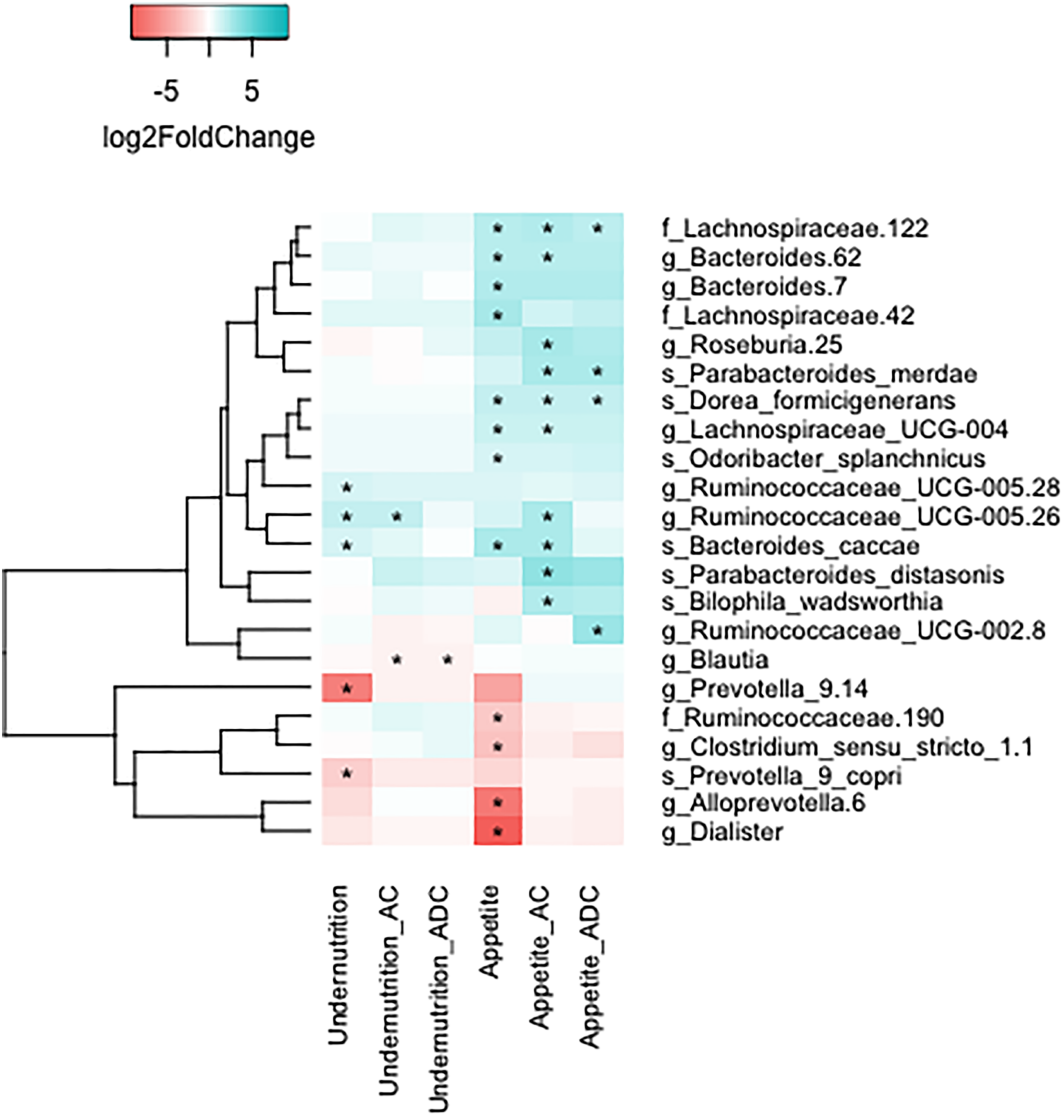
Log2 fold change of all bacterial taxa significantly associated with either poor appetite or undernutrition. Heatmap depicting the log2 fold change in bacterial abundance of taxa that are significantly associated with either undernutrition or poor appetite. Log2 fold change was calculated with DESeq models, either crude (first and fourth column), adjusted for age (second and fifth column), or adjusted for both age and Mediterranean Diet Score (third and sixth column). Blue cells depict a positive log2 fold change, indicating higher abundance in participants with undernutrition or poor appetite, whereas red cells depict a negative log2 fold change, indicating lower abundance in participants with undernutrition or poor appetite. Numbers behind taxa are arbitrary identifiers for clades within the specified rank. Asterisks indicate Benjamini–Hochberg‐adjusted *P*‐value < 0.01. Taxa were only considered if the mean abundance was >10. AC, age corrected; ADC, age and diet corrected.

Then, DESeq2 was used to evaluate differentially abundant bacterial taxa for poor appetite and undernutrition. These are depicted in *Figure* 2. Higher abundances of the family Lachnospiraceae, including *Dorea formicigenerans* , were found in participants with poor appetite, independent of age and MDS. This was also true for Ruminococcaceae UCG‐002 group, and *Parabacteroides merdae* . Likewise, a 0.67 times lower abundance of the genus *Blautia* of the Lachnospiraceae family was found in participants with undernutrition (log2 fold change = −0.57, Benjamini–Hochberg‐adjusted *P*‐value = 0.008). There was no overlap in the taxa that were associated with poor appetite and undernutrition after adjustment for both age and MDS.

### Faecal acetate levels are reduced in participants with either poor appetite or undernutrition

**Figure 3:**
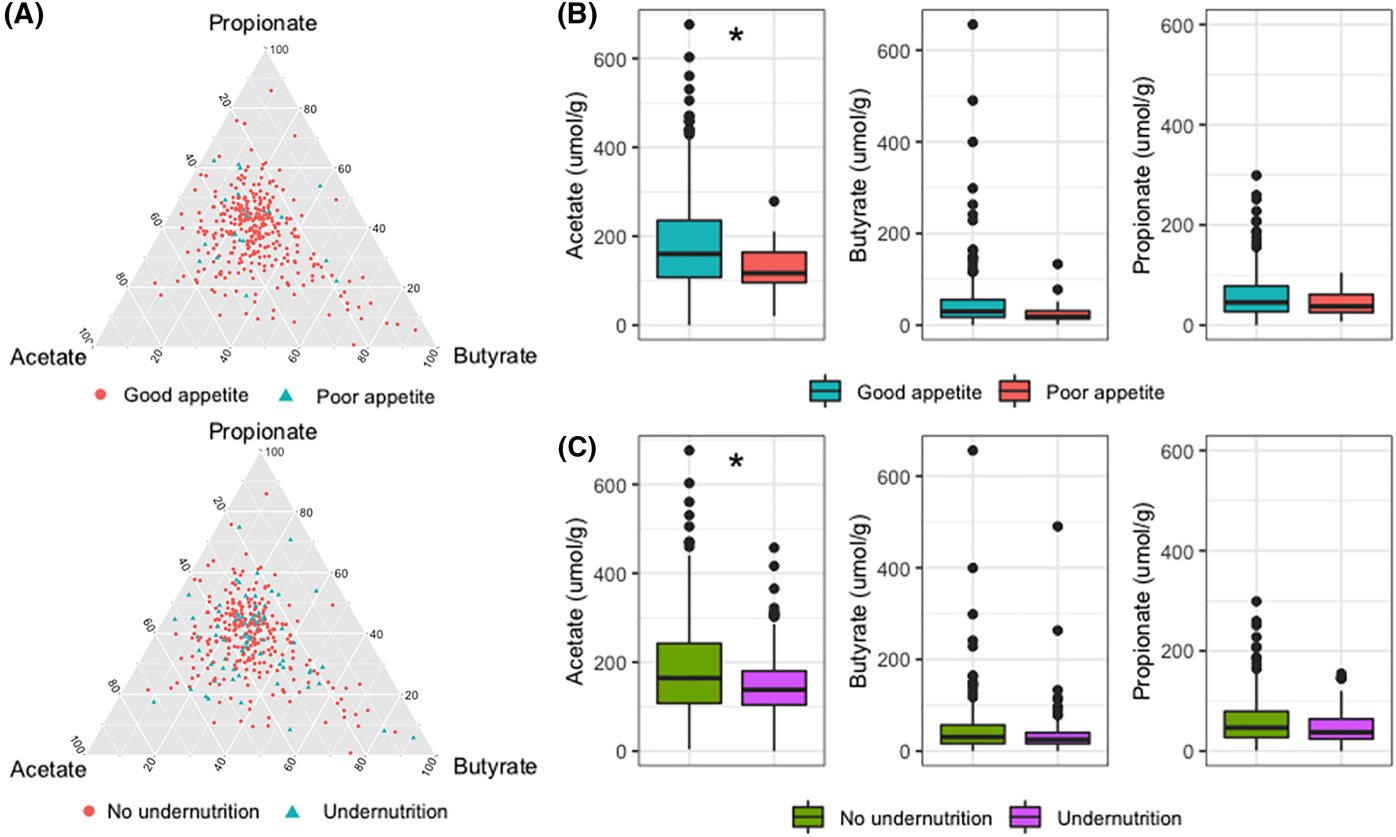
Associations of faecal short‐chain fatty acids with poor appetite and undernutrition. *(A)* Ternary diagrams showing faecal propionate, acetate, and butyrate as compositional data. Each point represents a participant with or without poor appetite and with or without undernutrition. The closer the participant is plotted to one of the short‐chain fatty acids, the higher the concentration of that short‐chain fatty acid is relative to the concentrations of the other short‐chain fatty acids. *(B* and *C)* Boxplots of faecal acetate, butyrate, and propionate concentrations for participants with or without poor appetite *(B)* and with or without undernutrition *(C)*. Boxplots show median (centre line), interquartile ranges (boxes), 1.5× the interquartile ranges (whiskers), and outliers. Differences were tested with Student's *t*‐test with Bonferroni correction.

We first performed metabolomics on plasma samples, which were collected in 149 participants. Of these participants, 43 were undernourished and 17 had poor appetite. A total of 961 metabolites were identified. Procrustes analysis showed that participants that had similar microbial composition also had more similar metabolite profiles (*P* < 0.00001). However, even though several microbial taxa were differentially abundant for poor appetite or undernutrition, none of the identified metabolites differed significantly between participants with or without poor appetite or undernutrition at a Benjamini–Hochberg‐adjusted *P*‐value < 0.01 (in crude or adjusted models). We then measured faecal SCFAs successfully in 344 participants. MANOVA showed that neither poor appetite nor undernutrition was associated with the SCFA composition (*Figure* 3A). However, absolute levels of faecal acetate were reduced in both poor appetite and undernutrition (Student's *t*‐test Bonferroni‐corrected *P*‐values 0.006 and 0.026, respectively; *Figure* 3B and 3C).

### Faecal microbiota from older adults with low body mass index tend to induce less weight gain in germ‐free mice

In five cages, mice had fought after colonization with subsequent wounding and possible consequences for body weight and composition (*Figure* S3). Accordingly, these cages and the corresponding donors were excluded from further analyses. Donor characteristics for the 12 remaining cages are presented in *Table* 1.

**Table 1:** Baseline characteristics mouse experiment

Characteristic	All	LBMI	WL	HC	*P*‐value
**Donors**	***n* = 12**	***n* = 5**	***n* = 4**	***n* = 3**	
Age (years)	78.6 [71.4 to 83.2]	75.9 [70.4 to 83.9]	80.9 [70.7 to 88.1]	79.4 [71.4 to 81.3]	0.871
Sex (male)	3 (25.0)	2 (40.0)	1 (25.0)	0 (0.0)	0.727
BMI (kg/m^2^)	23.5 [19.6 to 24.7]	19.4 [19.3 to 20.6]^a^	23.7 [23.4 to 24.5]	26.1 [24.6 to 26.6]^a^	0.010^a^
Weight difference (%/2 years)	−2.8 [−9.1 to −0.8]	−2.2 [−3.5 to 1.1]	−10.0 [−13.9 to −8.2]^a^	−0.7 [−1.1 to 2.0]^a^	0.016^a^
**Mice**	***n* = 41**	***n* = 17**	***n* = 14**	***N* = 10**	
Age (weeks)	10 [10 to 10]	10 [10 to 10]	10 [9 to 11]	10 [9 to 10]	0.060
Weight (g)	37.3 [35.4 to 39.3]	37.7 [36.7 to 40.3]^a^	37.5 [36.5 to 39.5]	35.1 [33.6 to 36.5]^a^	0.030^a^
Lean mass (g)	31.4 [29.6 to 32.7]	32.2 [31.1 to 33.7]^a^	31.4 [30.2 to 32.1]	29.7 [28.8 to 31.5]^a^	0.025^a^
Lean mass (%)	82.8 [80.1 to 85.6]	82.9 [79.7 to 87.3]	81.9 [79.7 to83.6]	85.4 [82.6 to 86.4]	0.181
Fat mass (g)	4.4 [3.6 to 5.2]	3.7 [3.2 to 5.0]	4.8 [4.3 to 5.8]	4.2 [3.6 to 5.6]	0.118
Fat mass (%)	11.9 [9.8 to 13.4]	9.9 [8.9 to 12.1]^a^	12.7 [11.6 to 14.7]^a^	12.0 [10.2 to 14.7]	0.024^a^

BMI, body mass index; HC, mice that received faecal microbiota from human donors without low BMI or substantial weight loss; LBMI, mice that received faecal microbiota from human donors with low BMI; WL, mice that received faecal microbiota from human donors with substantial weight loss.

Data are depicted in median [interquartile range] or number (percentage). Differences among groups are tested with Kruskal–Wallis test (continuous variables) or Fisher's exact test (categorical variables).

^a^ Groups that differ based on pairwise testing with Bonferroni correction.

**Figure 4:**
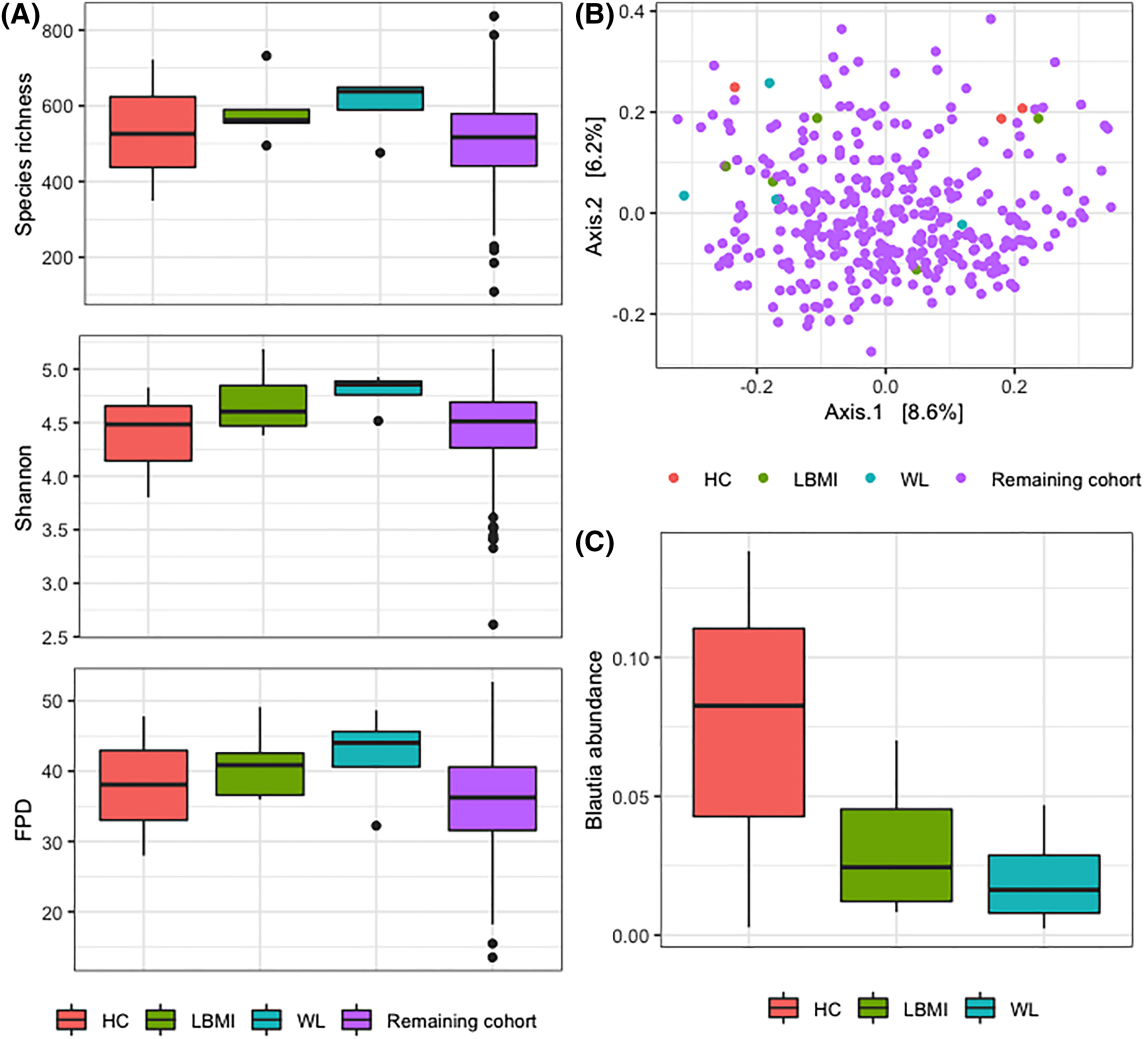
Alpha‐diversity, beta‐diversity, and *Blautia* of each of the donor groups. *(A)* Boxplots of species richness, Shannon diversity index, and FPD for each of the donor groups and the remaining cohort. Differences among groups were tested with Kruskal–Wallis tests. Boxplots show median (centre line), interquartile ranges (boxes), 1.5× the interquartile ranges (whiskers), and outliers. *(B)* Principal coordinate plot based on Bray–Curtis dissimilarity, coloured for each of the donor groups and the remaining cohort. Donors seem equally distributed throughout the cohort; donor group does not explain a significant amount of variance based on PERMANOVA. *(C)* Boxplot of *Blautia* abundance. There is no difference between donor groups based on Kruskal–Wallis test. Boxplots indicate same parameters as *(A)*. FPD, Faith's phylogenetic diversity; HC, mice that received faecal microbiota from human donors without low body mass index or substantial weight loss; LBMI, mice that received faecal microbiota from human donors with low body mass index; WL, mice that received faecal microbiota from human donors with substantial weight loss.

Alpha‐diversity did not differ among donor groups, nor did donor grouping explain a significant amount of variance in Bray–Curtis dissimilarity (*Figure* 4A and 4B). However, in line with the results from our cohort study, we observed that *Blautia* abundance was reduced in the LBMI and WL donors compared with the HC donors, although this was not statistically significant at *n* = 12 (*Figure* 4C). Engraftment of the transferred microbiota was considered successful as donor ID explained 90.7% of variation in microbiota composition among the Week 3 mouse samples, based on PERMANOVA (*P* < 0.001). Moreover, Week 3 faecal mouse microbiota differed significantly less from their corresponding donor compared with a random donor, based on Bray–Curtis dissimilarity.

**Figure 5:**
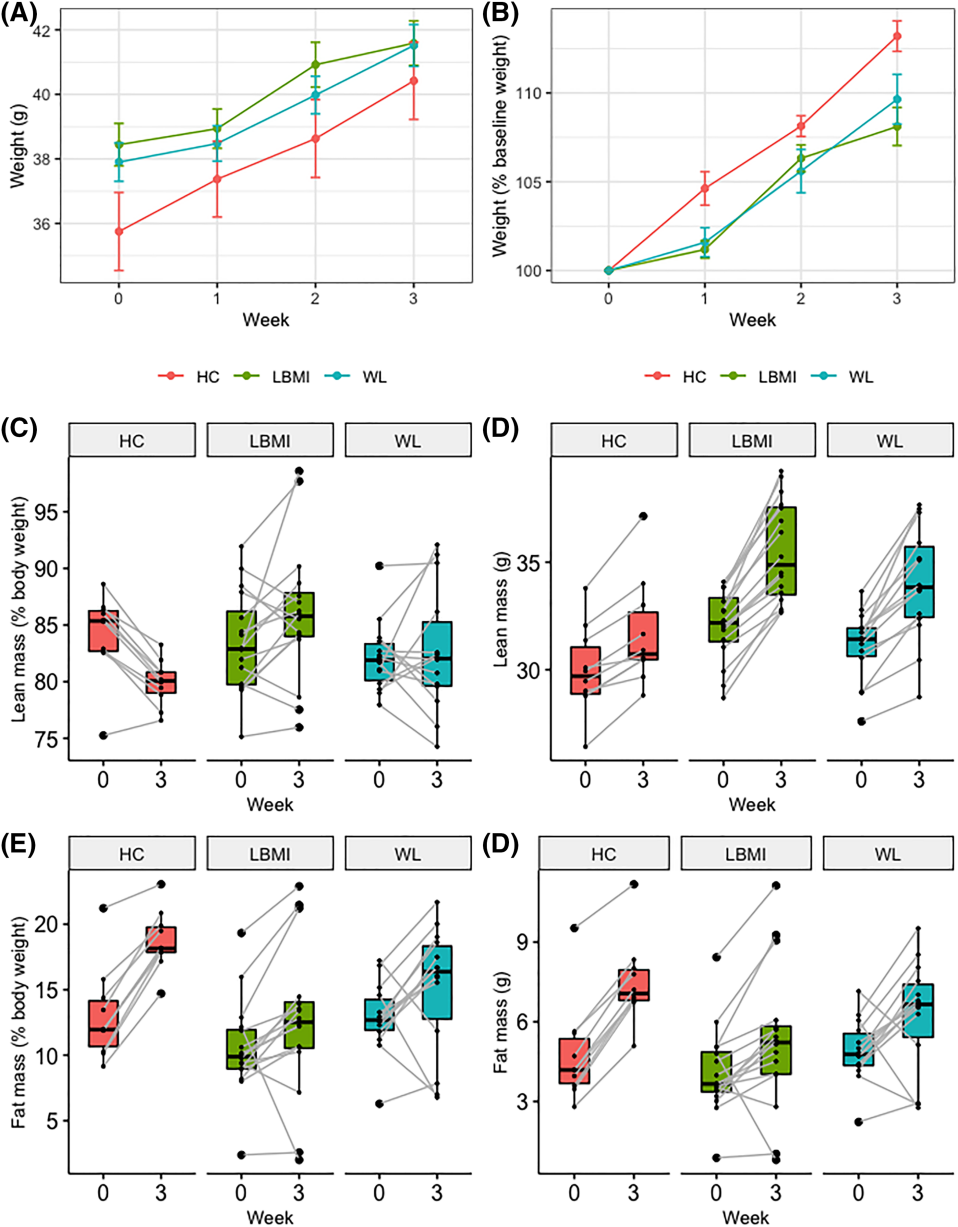
Weight, lean mass, and fat mass of mice during experiment. *(A* and *B)* Line graphs for average body weight in g *(A)*, and % of baseline weight *(B)* of mice per group during experiment (error bars indicate standard error). *(C* and *D)* Boxplots of lean mass in % of body weight *(C)* and g *(D)* per group at baseline and after a 3 week follow‐up. *(E* and *F)* Same as *(C)* and *(D)*, but for fat mass. Boxplot centre line indicates median, boxes indicate interquartile ranges, whiskers indicate 1.5× the interquartile ranges, and paired measurements are connected by grey lines. HC, mice that received faecal microbiota from human donors without low body mass index or substantial weight loss; LBMI, mice that received faecal microbiota from human donors with low body mass index; WL, mice that received faecal microbiota from human donors with substantial weight loss.

No significant differences in body weight, fat or lean mass, or food intake were observed among the groups of mice (*Figure* 5A–5F, *Table* 2). However, there was a trend for the LBMI mice to weigh 1.26 g less at Week 3 and have 6.13% more lean mass (in % body weight) compared with the HC mice (*P* = 0.086 and *P* = 0.067, respectively) (*Table* 2). Due to the exclusion of five cages, HC mice had lower baseline body weight and absolute lean mass than the LBMI mice. Therefore, all analyses were adjusted for baseline weight. Moreover, a sensitivity analysis in which we matched the three remaining HC cages to the three WL and LBMI cages with the lowest average baseline body weights (*n*_donors_ = 9, *n*_mice_ = 30) yielded similar results: the LBMI mice weighed 1.71 g less at Week 3 compared with the HC mice (*P* = 0.041). We concluded that the differences in baseline weight did not explain our findings.

**Table 2:** Differences in body weight, fat and lean mass, and food intake in LBMI vs. HC and WL vs. HC

	LBMI (*n*_donors_ = 5; *n*_mice_ = 17) vs. HC (*n*_donors_ = 3; *n*_mice_ = 10)			WL (*n*_donors_ = 4; *n*_mice_ = 14) vs. HC (*n*_donors_ = 3; *n*_mice_ = 10)		
	*B*	95% CI	*P*‐value	*B*	95% CI	*P*‐value
Overall body weight (g)	−0.76	−2.16 to 0.65	0.257	−0.69	−2.15 to 0.76	0.310
Body weight (g) at Week 1	−0.87	−2.33 to 0.60	0.226	−0.77	−2.29 to 0.75	0.292
Body weight (g) at Week 2	−0.14	−1.61 to 1.33	0.839	−0.53	−2.05 to 0.99	0.466
Body weight (g) at Week 3	−1.26	−2.73 to 0.21	0.086	−0.78	−2.30 to 0.74	0.286
Lean mass (g) at Week 3	1.36	−0.24 to 2.96	0.089	0.98	−0.67 to 2.63	0.214
Lean mass (%) at Week 3	6.13	−0.53 to 12.78	0.067	3.73	−3.23 to 10.68	0.257
Fat mass (g) at Week 3	−1.42	−3.98 to 1.13	0.241	−1.24	−3.90 to 1.42	0.320
Fat mass (%) at Week 3	−3.78	−9.56 to 2.00	0.174	−3.24	−9.24 to 2.76	0.254
Overall food intake (g/g)	0.06	−0.07 to 0.19	0.316	0.05	−0.08 to 0.18	0.422
Food intake (g/g) at Week 1	−0.03	−0.18 to 0.13	0.735	0.02	−0.15 to 0.18	0.838
Food intake (g/g) at Week 2	0.11	−0.05 to 0.27	0.153	0.07	−0.10 to 0.23	0.407
Food intake (g/g) at Week 3	0.09	−0.07 to 0.25	0.235	0.06	−0.10 to 0.23	0.425

HC, mice that received faecal microbiota from human donors without low body mass index or substantial weight loss; LBMI, mice that received faecal microbiota from human donors with low body mass index; WL, mice that received faecal microbiota from human donors with substantial weight loss.

Data are depicted in regression estimate (*B*), 95% confidence interval (95% CI), and *P*‐values. The number of donors and corresponding mice per group are reported. Differences between groups were tested with linear mixed models. Models for outcomes on body weight, lean mass, and fat mass models were adjusted for baseline values. Random intercepts for donor ID were included in all models to adjust for dependency of same‐donor colonization. For outcomes on body weight, random intercepts for mouse ID were included as well to adjust for repeated measures.

**Figure 6:**
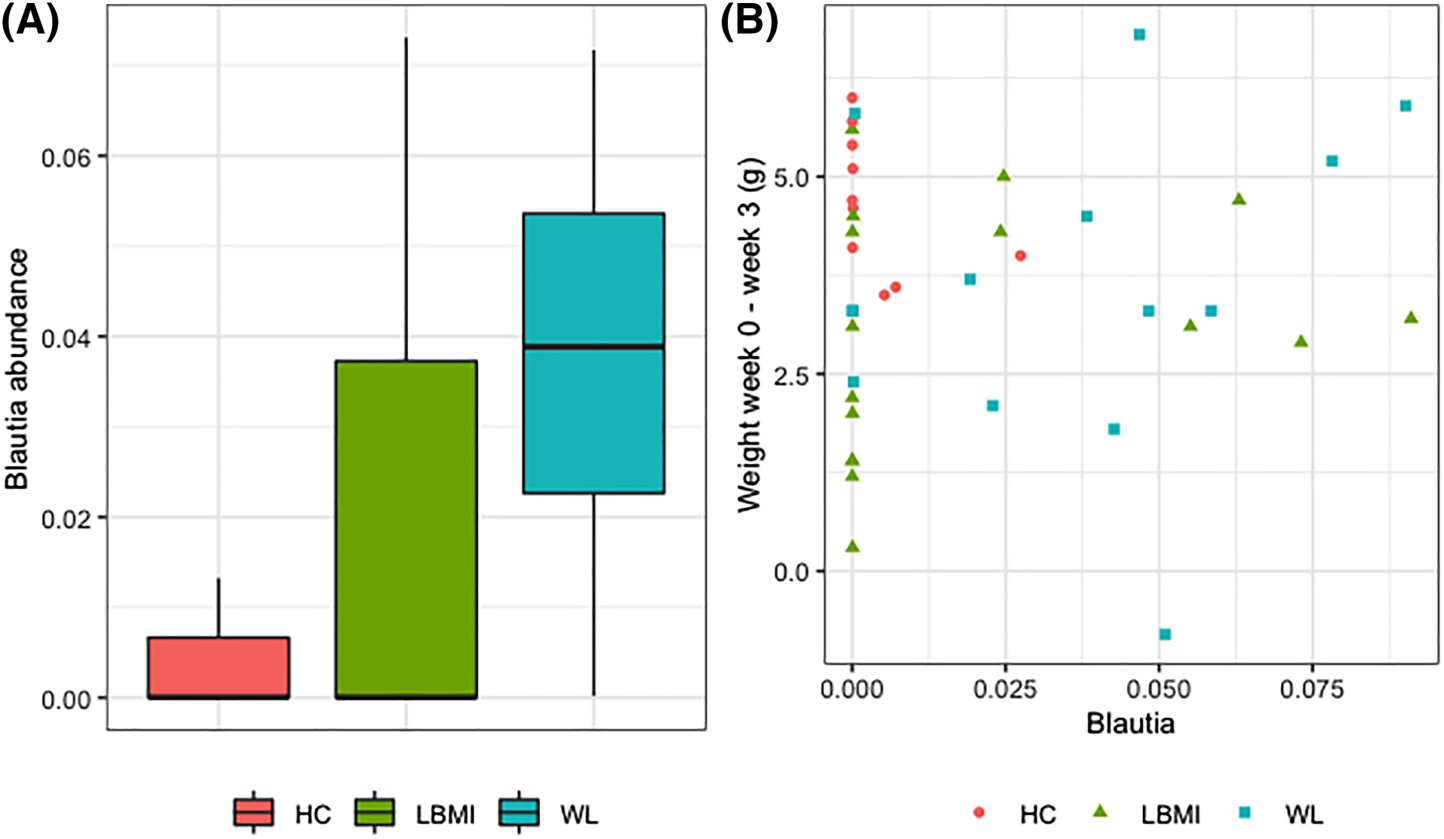
*Blautia* abundance in Week 3 mouse faecal samples. *(A)* Boxplot for *Blautia* abundance in Week 3 mouse faecal samples per group. *(B)* Scatterplot of *Blautia* abundance in Week 3 mouse faecal samples plotted against the weight difference in the mice from baseline to Week 3. Boxplot centre line indicates median, boxes indicate interquartile ranges, whiskers indicate 1.5× the interquartile ranges, and paired measurements are connected by grey lines. HC, mice that received faecal microbiota from human donors without low body mass index or substantial weight loss; LBMI, mice that received faecal microbiota from human donors with low body mass index; WL, mice that received faecal microbiota from human donors with substantial weight loss.

Finally, we explored whether the 3 week faecal microbiota of the mice was related to mouse phenotypes. Body weight, body composition, or food intake explained a significant amount of variance in Bray–Curtis dissimilarity (PERMANOVA *P* > 0.1). Finally, we studied the *Blautia* taxon that was identified in the human cohort. It engrafted well as it was the most abundant taxon in the mouse samples, but was not present in every cage (*Figure* 6B). Nonetheless, the trend of reduced *Blautia* that was observed in LBMI and WL donors was not reproduced in the mouse samples (*Figure* 6A). Moreover, *Blautia* abundance was not related to weight change from baseline to Week 3 in the mice (*Figure* 6B).

## Discussion

We found several associations of the gut microbiota with both poor appetite and undernutrition, pertaining to overall microbiota composition (i.e. alpha‐diversity and beta‐diversity) and specific bacterial species. Moreover, participants with poor appetite or undernutrition had reduced levels of faecal acetate. Finally, a faecal microbiota transfer from participants with an LBMI tended to induce less weight gain in germ‐free mice.

Our research first focused on the overall microbiota composition (i.e. Bray–Curtis dissimilarity). In line with literature,^8^ diet (operationalized as MDS) and fibre intake were the most important determinants. The other microbiota determinants we found (e.g. age, BMI, cognitive functioning, sex, smoking, HbA1c, Hb, and blood pressure) have also previously been described.^8^, ^35^ Whereas the association of poor appetite with Bray–Curtis dissimilarity was MDS dependent, we demonstrated increased abundances of four taxa for participants with poor appetite, independent of possible confounders. Recently, Cox *et al*.^4^ also demonstrated a microbiota–appetite relationship in older adults that was diet independent.^4^ They reported lower diversity and lower abundance of several Lachnospiraceae taxa in participants with poor appetite,^4^ whereas we found no association with alpha‐diversity and higher abundance in several Lachnospiraceae taxa. These inter‐study discrepancies could be explained by differences in study design, or by the time lag of up to 8 years between microbiota sampling, dietary data collection, and appetite assessment in the study of Cox *et al*.^4^ The higher abundance of Lachnospiraceae in our participants with poor appetite was in line with a study in Mexican children where higher Lachnospiraceae abundance correlated negatively with energy intake and positively with leptin levels.^36^ Generally, Lachnospiraceae taxa are associated with high SCFA production and energy extraction from the diet.^37^ With regard to poor appetite, these taxa were hypothesized to adapt to poor nutrition and protect from the development of undernutrition.^36^

Undernourished participants had higher alpha‐diversity, irrespective of age. This is an interesting finding, since undernutrition‐associated conditions sarcopenia^9^ and frailty^10^ are generally found to be associated with lower alpha‐diversity. This is, however, also true for conditions such as obesity^38^ and weight gain.^39^ Although we excluded participants with obesity and substantial weight gain, well‐nourished participants approaching these conditions could have driven the lower alpha‐diversity in the participants without undernutrition. Undernutrition was associated with a lower abundance of the genus *Blautia*. *Blautia* belongs to the Lachnospiraceae family and is being investigated for its probiotic properties.^40^ It is thought to prevent the colonization of pathogens by producing bacteriocins^40^ and exhibits anti‐inflammatory properties by up‐regulating regulatory T cells and SCFA production.^37^ Theoretically, reduced *Blautia* abundance could increase chronic low‐grade inflammation and decrease energy uptake from the diet by fermentation. Further research is needed to replicate our results, but prebiotics or probiotics aimed at increasing the abundance of *Blautia* may be of interest in the prevention of undernutrition in older adults.

In our cohort, participants with either poor appetite or undernutrition had significantly lower faecal acetate levels. Lower acetate levels in faeces of undernourished participants are in line with a reduced abundance of *Blautia*, which is a potent acetate producer.^37^, ^40^ It suggests reduced energy extraction from the diet. The reduced acetate levels in participants with poor appetite contradict studies suggesting a satietogenic effect of SCFAs.^41^ Possibly, this satietogenic effect is blunted or absent in older adults due to age‐related changes in physiology, as was suggested for the satietogenic effect of protein.^42^ Nevertheless, it must be noted that faecal SCFA levels are not a direct measure of intestinal SCFA concentrations but rather a net result of SCFA production after subtracting intestinal SCFA absorption and microbial cross‐feeding.^5^ Finally, we showed that the plasma metabolite profiles were not associated with poor appetite or undernutrition. Possibly, this is due to the smaller population in which we measured plasma metabolites.

To evaluate causality, we conducted a faecal microbiota transfer experiment in germ‐free mice. In our experiment, alpha‐diversity and beta‐diversity did not differ among donor groups. Nevertheless, the lack of compositional differences between donor groups does not necessarily reflect functional resemblance. Functional redundancy between species as well as functional variations within species is common among bacteria. LBMI mice, but not WL mice, tended to gain less weight than HC mice, but relatively more lean mass. These group differences do not seem to be caused by baseline differences between LBMI and HC mice as we adjusted our analyses for baseline values and conducted a sensitivity analysis in which we matched three cages per group based on baseline body weight. The effects could also not be attributed to differences in food intake, which suggests that undernutrition‐associated microbiota might indeed affect host energy extraction or metabolism. Possibly, the LBMI‐associated gut microbiota interact with physiological mechanisms aimed at the preservation of lean mass in older adults. Our finding is in agreement with a previous study assessing the role of the gut microbiota in Malawian children with Kwashiorkor, where undernutrition‐associated microbiota also induced less weight gain.^43^ However, our results must be interpreted with caution as the trend we show was not statistically significant. We could not demonstrate an association of the faecal mouse microbiota at Week 3 with mouse phenotype, but this analysis was possibly underpowered because we only included one random mouse per donor (*n* = 12). Therefore, more experiments are needed to replicate our results and further elucidate the mechanisms by which aged, undernutrition‐associated microbiota might affect its host.

### Strengths and limitations

We studied a rather large cohort of older adults and evaluated a host–microbiota relation in a very comprehensive manner, including associations with overall microbiota composition, specific taxa, and with plasma and faecal metabolites. However, our population was relatively vital and poor appetite and undernutrition occurred in a small proportion of participants (5.9% and 21.5%, respectively). Possibly, the associations with the gut microbiota would be more pronounced in a less vital, institutionalized population, as has been suggested before.^8^ We did not conduct full metagenomics of the microbiota and could therefore not directly assess its full metabolic capacity. The use of mouse models comes with some inherent limitations.^34^ Most importantly, human and mouse (patho)physiology and living conditions differ substantially. This impedes full colonization of the human‐associated microbiota and may affect the microbiota–host interactions. However, human faecal transfer experiments are ethically not an option when evaluating potentially harmful effects of microbial communities in vulnerable elderly individuals.^34^ Finally, although we performed the mouse experiment as first step in assessing causality, future longitudinal studies are needed to demonstrate a temporal relationship between the microbiota, poor appetite, and undernutrition in humans.

## Conclusions

The age‐related reduction in appetite is considered the most important risk factor for the development of undernutrition in older adults.^3^ In our cohort of community‐dwelling older adults, we demonstrated that the gut microbiota is related to both poor appetite and undernutrition. Irrespective of age and diet, increased abundances of *Lachnospiraceae*, *Ruminococcaceae* UCG‐002, *P. merdae* , and *D. formicigenerans* were found in participants with poor appetite, whereas reduced abundance of *Blautia* was found in participants with undernutrition. Both poor appetite and undernutrition were associated with reduced levels of faecal acetate. Finally, there was a trend of faecal microbiota from older adults with LBMI to induce less weight and more lean mass gain in germ‐free mice than faecal microbiota from older adults without an LBMI. Possibly, microbiota‐manipulating strategies will benefit older adults prone to undernutrition.

## Funding

This study was supported by the European Union Horizon 2020 PROMISS project ‘PRevention Of Malnutrition In Senior Subjects in the EU’ (grant agreement no. 678732). The content only reflects the author's view and the Commission is not responsible for any use that may be made of the information it contains. The Longitudinal Aging Study Amsterdam is supported by a grant from the Netherlands Ministry of Health Welfare and Sports, Directorate of Long‐Term Care. The LASA data collection in 2012–13 was financially supported by the Netherlands Organization for Scientific Research (NWO) in the framework of the project ‘New Cohorts of young old in the 21st century’ (file number 480‐10‐014). M.N. is supported by a personal ZONMW VIDI grant 2013 (016.146.327), a personal ZONMW VICI grant 2020 (09150182010020), and a Dutch Heart Foundation CVON IN CONTROL Young Talent Grant 2013. F.B. and M.N. are supported by a Transatlantic Networks of Excellence Award from the Leducq Foundation (17CVD01) and from JPI A Healthy Diet for a Healthy Life (2017‐01996_3).

## Conflict of interests

The authors declare no competing interests.

## Supporting information


**Table S1** **Participant characteristics of the human cohort**
**Figure S1 Abundance of microbial phyla and genera, stratified to appetite and nutritional status.** Relative abundance of microbial phyla stratified to appetite (A), and nutritional status (B), and relative abundance of 20 most abundant genera stratified to appetite (C), and nutritional status (D).
**Figure S2 Correlogram of all variables that explain a significant amount of Bray‐Curtis distance.** A correlogram depicting the correlations among all variables that explain a significant amount of Bray‐Curtis distance. The heatmap depicts the regression coefficient of statistically significant correlations (Spearman p‐value<0.05). Blue indicates a positive correlation and red a negative correlation. MDS: Mediterranean diet score; BMI: body mass index; MMSE: mini‐mental state examination; CESD: Centre for Epidemiologic Studies Depression.
**Figure S3 Body weight of each individual mouse during experiment stratified by cages.** The body weight in g (A) and % of baseline weight (B) stratified by cages during the experiment. Scarring and wounds were observerd on mice in cages 5, 6, 7, 9, and 14. All mice in cage 5 were euthanized after 1 week due to the severity of their wounds, this was also true for 1 mouse from cage 7. One mouse from cage 1 was found dead on day 3 of the experiment, likely due to a complication from the gavage.
