# Supplementary material for: Gut microbial characteristics in poor appetite and undernutrition: a cohort of older adults and microbiota transfer in germ‐free mice
Source: J Cachexia Sarcopenia Muscle. 2022 Jun 14;13(4):2188–201. doi: 10.1002/jcsm.13002 (PMC9397553; doi:10.1002/jcsm.13002)
Supplement: Supplementary file 1 — Table S1 Participant characteristics of the human cohort Figure S1 Abundance of microbial phyla and genera, stratified to appetite and nutritional status. Relative abundance of microbial phyla stratified to appetite (A), and nutritional status (B), and relative abundance of 20 most abundant genera stratified to appetite (C), and nutritional status (D). Figure S2 Correlogram of all variables that explain a significant amount of Bray‐Curtis distance. A correlogram depicting the correlations among all variables that explain a significant amount of Bray‐Curtis distance. The heatmap depicts the regression coefficient of statistically significant correlations (Spearman p‐value<0.05). Blue indicates a positive correlation and red a negative correlation. MDS: Mediterranean diet score; BMI: body mass index; MMSE: mini‐mental state examination; CESD: Centre for Epidemiologic Studies Depression. Figure S3 Body weight of each individual mouse during experiment stratified by cages. The body weight in g (A) and % of baseline weight (B) stratified by cages during the experiment. Scarring and wounds were observerd on mice in cages 5, 6, 7, 9, and 14. All mice in cage 5 were euthanized after 1 week due to the severity of their wounds, this was also true for 1 mouse from cage 7. One mouse from cage 1 was found dead on day 3 of the experiment, likely due to a complication from the gavage. [file JCSM-13-2188-s001.docx]

**Table S1 Participant characteristics of the human cohort**

| **Characteristics** | **n** | **All** | **Poor appetite (n=21)** | **Normal appetite (n=336)** | **Undernutrition (n=77)** | **No undernutrition (n=281)** |
| --- | --- | --- | --- | --- | --- | --- |
| ***Demographics*** |  |  |  |  |  |  |
| Age (yrs) | 358 | 73 [69-77] | 77 [72-83] | 73 [69-77] | 74 [70-80] | 72 [69-76] |
| Sex (male) | 358 | 209 (58.4) | 9 (42.9) | 199 (59.2) | 30 (39.0) | 179 (63.7) |
| ***Appetite and undernutrition*** |  |  |  |  |  |  |
| Undernutrition^a^ | 358 | 77 (21.5) | 9 (42.9) | 68 (20.2) | 77 (100.0) | 281 (100.0) |
| Low BMI^b^ |  | 40 (11.2) | 5 (23.8) | 35 (10.4) | 40 (51.9) | 281 (100.0) |
| Weight loss^c^ |  | 43 (12.0) | 4 (19.0) | 39 (11.6) | 43 (55.8) | 281 (100.0) |
| Appetite (CNAQ score) | 357 | 31 [30-33] | 24 [21-26] | 31 [30-33] | 31 [30-32] | 31 [30-33] |
| Poor appetite^d^ |  | 21 (5.9) | 21 (100.0) | 0 (0.0) | 9 (11.7) | 12 (4.3) |
| ***Body measurements*** |  |  |  |  |  |  |
| BMI (kg/m^2^) | 358 | 24.8 ± 2.5 | 24.5 ± 3.0 | 24.8 ± 2.5 | 22.7 ± 2.3 | 25.3 ± 2.3 |
| Weight (kg) | 358 | 75.6 ± 10.9 | 71.4 ± 0.3 | 74.8 ± 11.0 | 67.2 ± 10.0 | 76.6 ± 10.3 |
| Weight difference (%bodyweight/2yrs) | 358 | -0.5 ± 4.5 | 0.2 ± 7.5 | -0.5 ± 4.2 | -4.0 ± 5.7 | 0.5 ± 3.5 |
| ASMMI (kg/m^2^) | 343 | 6.8 ± 0.9 | 6.3 ± 0.9 | 6.8 ± 0.8 | 6.2 ± 0.7 | 7.0 ± 0.8 |
| Calf Circumference | 357 | 36.8 ± 2.5 | 35.7 ± 2.4 | 36.9 ± 2.5 | 35.6 ± 2.3 | 37.2 ± 2.5 |
| Mid Upper Arm Circumference | 358 | 31.1 ± 2.5 | 30.6 ± 3.2 | 31.1 ± 2.4 | 29.8 ± 2.7 | 31.4 ± 2.3 |
| Systolic Blood Pressure (mmHg) | 357 | 138.9 ± 19.0 | 136.2 ± 16.1 | 139.0 ± 19.2 | 133.2 ± 19.3 | 140.4 ± 18.7 |
| Diastolic Blood Pressure (mmHg) | 357 | 81.1 ± 10.3 | 82.2 ± .0 | 81.1 ± 10.4 | 77.6 ± 10.4 | 82.1 ± 10.1 |
| Pulse (bpm) | 357 | 66.2 ± 9.9 | 71.3 ± 6.9 | 65.9 ± 9.9 | 65.4 ± 9.7 | 66.4 ± 10.0 |
| ***Food intake*** |  |  |  |  |  |  |
| Calory intake (kcal/day) | 355 | 2116.8 ± 530.1 | 1961.0 ± 598.9 | 2128.0 ± 525.2 | 2143 ± 591.2 | 2110 ± 513.6 |
| Carbohydrate intake (EN%) | 355 | 41.1 ± 6.7 | 43.8 ± 8.5 | 41.0 ± 6.6 | 41.0 ± 7.2 | 41.2 ± 6.6 |
| Protein intake (EN%) | 355 | 14.6 ± 2.3 | 13.2 ± 2.6 | 14.7 ± 2.2 | 14.7 ± 2.6 | 14.6 ± 2.2 |
| **Fat intake** (EN%) | 355 | 34.9 ± 5.7 | 35.9 ± 5.9 | 34.8 ± 5.7 | 35.9 ± 6.2 | 34.6 ± 5.6 |
| Alcohol intake (EN%) | 355 | 3.1 [0.6-6.9] | 0.6 [0.1-3.4] | 3.1 [0.7-7.1] | 3.0 [0.4-4.9] | 3.1 [0.6-7.2] |
| Fibre intake (**g/**MJ) | 355 | 2.6 ± 0.6 | 2.1 ± 0.6 | 2.6 ± 0.6 | 2.5 ± 0.7 | 2.6 ± 0.6 |
| Mediteranean Diet Score | 355 | 33.0 ± 4.6 | 28.1 ± 5.1 | 33.3 ± 4.4 | 32.4 ± 4.9 | 33.2 ± 4.5 |
| ***Sensory function*** |  |  |  |  |  |  |
| Self-reported poor smell | 358 | 21 (5.9) | 4 (19.0) | 17 (5.1) | 5 (6.5) | 16 (5.7) |
| Measured poor smell | 357 | 65 (18.2) | 6 (28.6) | 59 (17.6) | 19 (24.7) | 46 (16.4) |
| Self-reported poor taste | 358 | 10 (2.8) | 5 (23.8) | 5 (1.5) | 4 (0.5) | 6 (2.1) |
| Measured poor taste | 357 | 32 (9.0) | 1 (4.8) | 31 (9.3) | 6 (0.8) | 26 (9.3) |
| ***Covariates*** |  |  |  |  |  |  |
| MMSE-score | 358 | 29 [28-30] | 27 [26-28] | 29 [28-30] | 29 [27-30] | 29 [28-29] |
| CESD-score | 356 | 4 [2-8] | 14 [8-16] | 4 [2-8] | 6 [3-9] | 4 [2-8] |
| Smoking | 358 |  |  |  |  |  |
| Never |  | 99 (27.7) | 4 (19.0) | 95 (28.3) | 26 (33.8) | 73 (26.0) |
| Former |  | 232 (64.8) | 11 (52.4) | 22 (65.5) | 44 (57.1) | 188 (66.9) |
| Current |  | 27 (7.5) | 6 (28.6) | 21 (6.2) | 7 (9.1) | 20 (7.1) |
| Medication | 358 |  |  |  |  |  |
| 0 drugs |  | 98 (27.4) | 4 (19.0) | 94 (28.0) | 24 (31.2) | 74 (26.3) |
| 1-4 drugs |  | 190 (53.1) | 8 (38.1) | 181 (53.9) | 33 (42.9) | 157 (55.9) |
| ≥ 5 drugs |  | 70 (19.6) | 9 (42.9) | 61 (18.2) | 20 (26.0) | 50 (17.8) |
| Income | 341 |  |  |  |  |  |
| Low |  | 102 (29.9) | 13 (72.2) | 89 (27.6) | 30 (40.5) | 72 (27.0) |
| Moderate |  | 121 (35.5) | 5 (27.8) | 116 (36.0) | 27 (36.5) | 94 (35.2) |
| High |  | 118 (34.6) | 0 (0.0) | 117 (36.3) | 17(23.0) | 101 (17.8) |
| Education | 358 |  |  |  |  |  |
| Low |  | 38 (10.6) | 4 (19.0) | 34 (10.1) | 7 (9.1) | 31 (11.0) |
| Medium |  | 187 (52.2) | 15 (71.4) | 171 (50.9) | 47 (61.0) | 140 (49.8) |
| High |  | 133 (37.2) | 2 (0.1) | 131 (39.0) | 23 (29.9) | 110 (39.1) |
| ***Biochemistry*** |  |  |  |  |  |  |
| HbA1c (%) | 148 | 5.7 [5.5 – 6.0] | 5.7 [5.7-6.0] | 5.7 [5.5-6.0] | 5.7 [5.5-6.0] | 5.7 [5.5-6.0] |
| Hb (mmol/l) | 148 | 8.6 ± 0.8 | 8.6 ± 0.7 | 8.6 ± 0.8 | 8.4 ± 0.6 | 8.7 ± 0.8 |
| Leucocytes (mmol/l) | 148 | 6.2 ± 1.5 | 6.1 ± 1.7 | 6.2 ± 1.5 | 6.2 ± 1.4 | 6.2 ± 1.6 |
| Fecal acetate (umol/g) | 344 | 159.3 [104.8-230.8] | 117.0 [95.7-163.9] | 160.3 [107.4-235.6] | 138.0 [103.9-180.4] | 164.3 [107.6-242.4] |
| Fecal butyrate (umol/g) | 344 | 29.0 [16.6-55.2] | 18.4 [13.9-31.2] | 29.8 [17.2-55.7] | 25.1 [16.5-40.2] | 30.7 [16.7-57.0] |
| Fecal propionate (umol/g) | 344 | 45.1 [26.6-76.7] | 37.5 [25.4-61.3] | 45.5 [26.9-78.2] | 37.5 [24.3-64.3] | 46.7 [27.2-79.4] |
| ***Sampling*** |  |  |  |  |  |  |
| Sample season | 358 |  |  |  |  |  |
| Summer |  | 69 (19.3) | 5 (23.8) | 63 (18.8) | 21 (27.3) | 48 (17.1) |
| Fall |  | 80 (22.3) | 10 (47.6) | 70 (20.8) | 20 (26.0) | 60 (21.4) |
| Winter |  | 86 (24.0) | 3 (14.3) | 83 (24.7) | 18 (23.4) | 68 (24.2) |
| Spring |  | 123 (34.4) | 3 (14.3) | 20 (35.7) | 18 (23.4) | 105 (37.4) |
| Time to freezer (hours) | 331 | 28 [18-32] | 29 [22-31] | 28 [18-32] | 27 [14-32] | 28 [19-32] |

Data is depicted in mean ± standard deviation, median [interquartile range], or number (percentage).

BMI: Body Mass Index; CNAQ: Council of Nutrition Appetite Questionnaire; ASMMI: Appendicular Skeletal Muscle Mass Index; Kcal: kilocalorie; EN%: percentage energy intake; MJ: megajoule; MMSE: Mini Mental State Exam; CESD: Center of Epidemiological Studies Depression.

^a^>5% bodyweight loss averaged over 2 years or BMI<20 (if age <70) or BMI<22 (if age ≥ 70); ^b^BMI < 20 (if age <70) or < 22 (if age ≥ 70); ^c^Weight loss >5% bodyweight/2 years; ^d^CNAQ-score <28


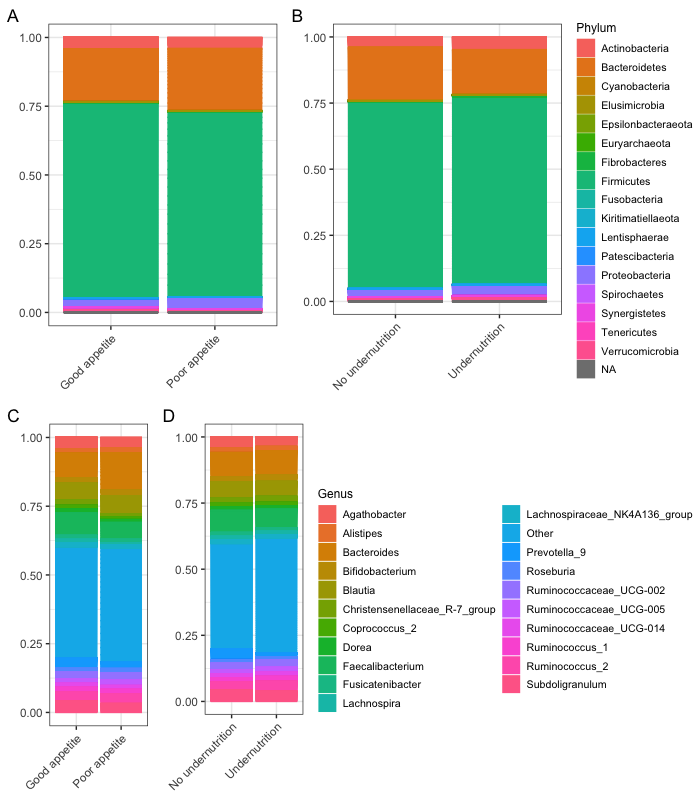


**Figure S1 Abundance of microbial phyla and genera, stratified to appetite and nutritional status**

Relative abundance of microbial phyla stratified to appetite (A), and nutritional status (B), and relative abundance of 20 most abundant genera stratified to appetite (C), and nutritional status (D).


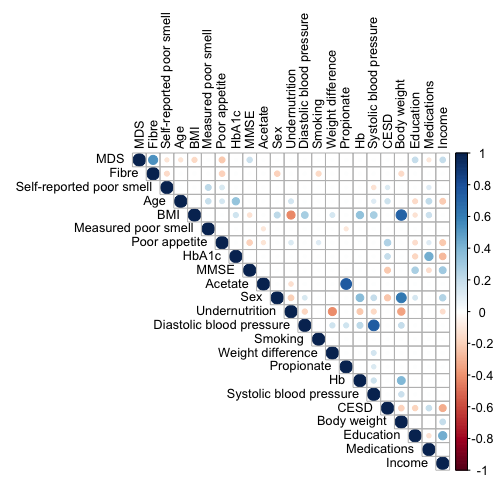


**Figure S2 Correlogram of all variables that explain a significant amount of Bray-Curtis distance**

A correlogram depicting the correlations among all variables that explain a significant amount of Bray-Curtis distance. The heatmap depicts the regression coefficient of statistically significant correlations (Spearman p-value<0.05). Blue indicates a positive correlation and red a negative correlation. MDS: Mediterranean diet score; BMI: body mass index; MMSE: mini-mental state examination; CESD: Centre for Epidemiologic Studies Depression.


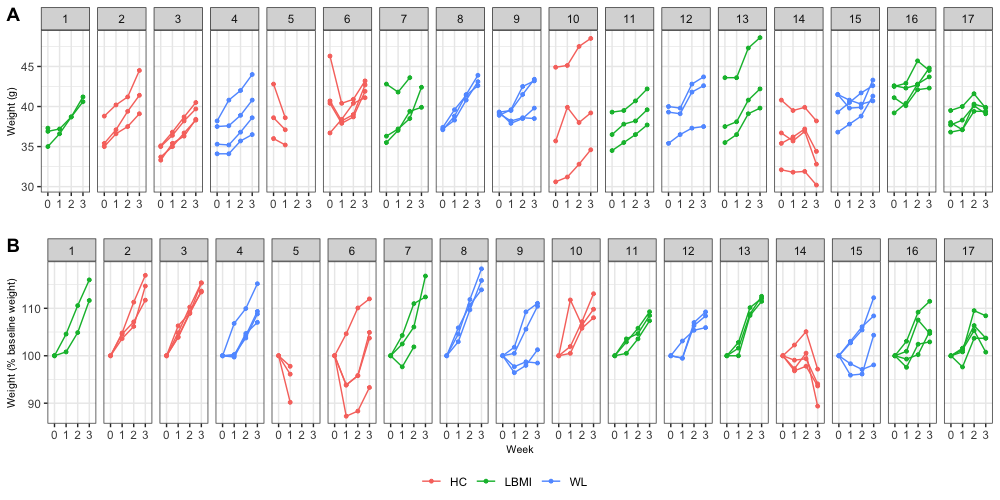


**Figure S3 Body weight of each individual mouse during experiment stratified by cages.**

The body weight in g (A) and % of baseline weight (B) stratified by cages during the experiment. Scarring and wounds were observerd on mice in cages 5, 6, 7, 9, and 14. All mice in cage 5 were euthanized after 1 week due to the severity of their wounds, this was also true for 1 mouse from cage 7. One mouse from cage 1 was found dead on day 3 of the experiment, likely due to a complication from the gavage.
